# Supplementary material for: Skin color-specific and spectrally-selective naked-eye dosimetry of UVA, B and C radiations
Source: Nat Commun. 2018 Sep 25;9:3743. doi: 10.1038/s41467-018-06273-3 (PMC6156228; doi:10.1038/s41467-018-06273-3)
Supplement: Supplementary file 1 — Supplementary Information [file 41467_2018_6273_MOESM1_ESM.pdf]

SUPPLEMENTARY INFORMATION

Skin color-specific and spectrally-selective naked-eye dosimetry of UVA, B and C radiations

Zou *et al.*

Skin type		I	II	III	IV	V	VI
Skin color		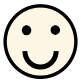	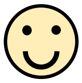	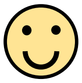	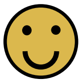	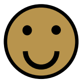	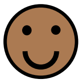
		Very fair	Fair	Medium	Olive	Brown	Dark brown
MED	UVB ($J{\bullet}m^{-2}$)	200-300	250-350	300-500	450-600	600-1000	1000-2000
	UVA ($J{\bullet}m^{-2}$)	200-350 ($x10^3$)	300-450 ($x10^3$)	400-550 ($x10^3$)	500-800 ($x10^3$)	700-1000 ($x10^3$)	>1000 ($x10^3$)

Supplementary Table 1. *UVB and UVA minimal erythemal doses (MED) for different skin types. MED is defined as the lowest threshold dose that may produce sunburn. The MED distinctly depends upon the skin sensitivity of a person, and as such, based on MED, the skin has been classified on the Fitzpatrick scale, ranging from type I (very fair) to type VI (dark brown). Further, the MED for UVA and UVB are different for each of the skin types. Since UVA has lower energy than UVB, the MED for UVA is approximately three orders of magnitude higher than that for UVB.*

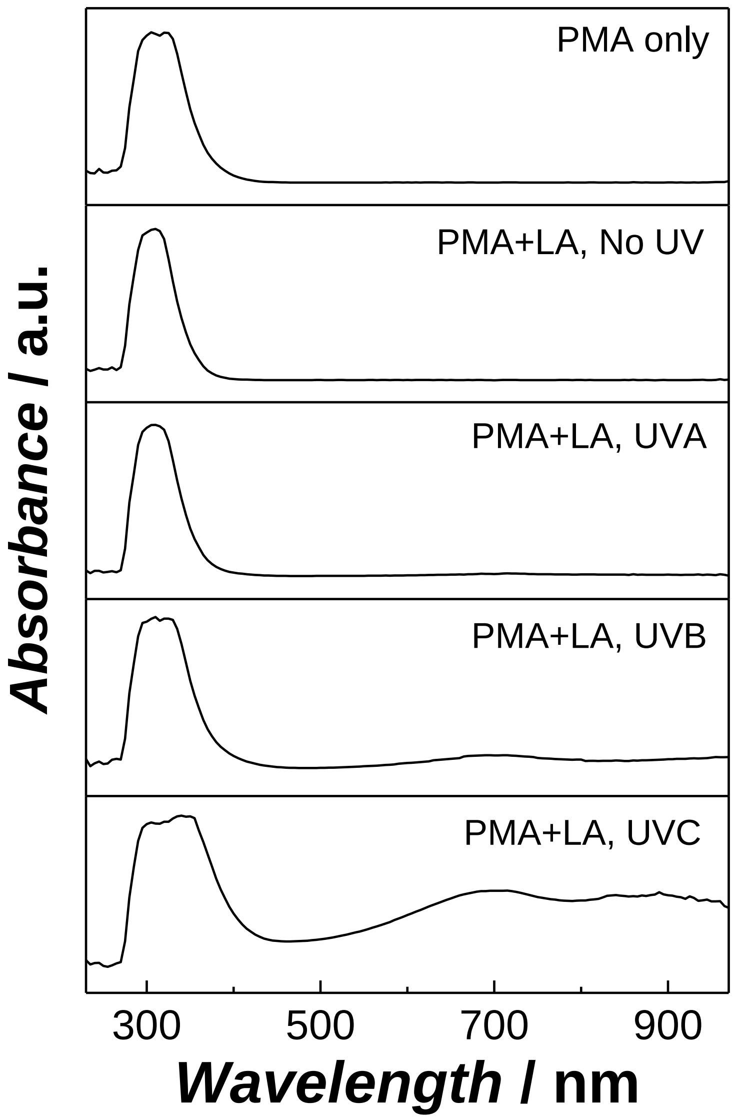

Supplementary Fig. 1 *UV-visible absorbance spectra of aqueous solutions of PMA, and PMA-LA mixtures before and after photoexcitation with UVA, B and C for 30 min with an intensity of 15 W·m^{-2}, corresponding to an overall dose of* 27,000 J·m^{-2} over 30 min at the sample surface.

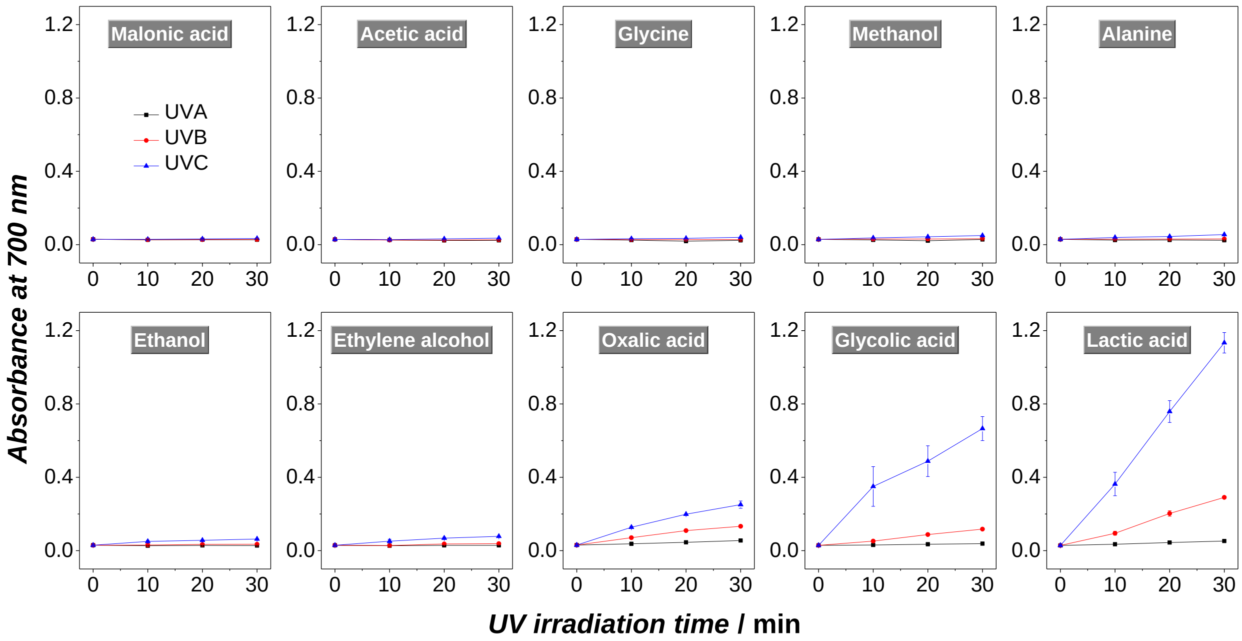

Supplementary Fig. 2 *UVR exposure time-dependent response of PMA in the presence of 10 different electron donors, as obtained from the optical absorbance at 700 nm. The final concentrations of PMA and electron donors correspond to 1 mM and 10 mM, respectively. The UVR intensity corresponds to 15 W·m*$^{-2}$*. Each data point represents an average of the colorimetric response obtained from 3 independent samples and the associated standard deviation.*

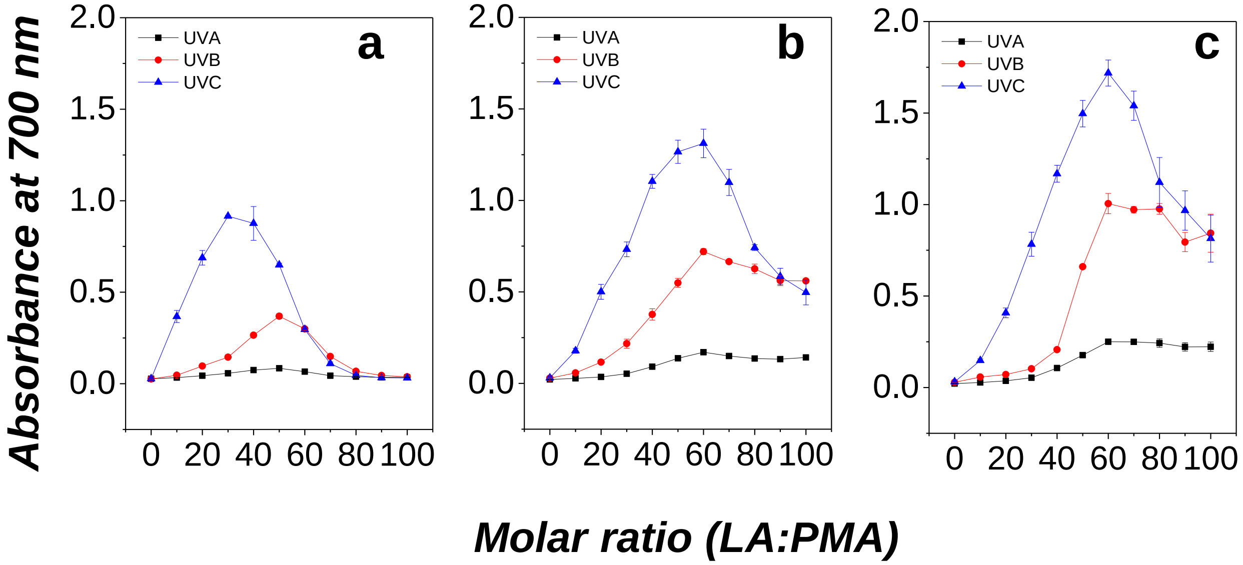

Supplementary Fig. 3 *Influence of the concentration of PMA (a) 1 mM, (b) 2.5 mM, and (c) 5 mM, and LA:PMA Molar ratios on sensor response measured from the absorbance at 700 nm after exposing samples for 5 min with a UV dose of 4,500 J·m^{-2}. The UVR intensity at the sample surface was 15 W·m^{-2}. Each data point represents an average of the colorimetric response obtained from 3 independent samples and the associated standard deviation.*

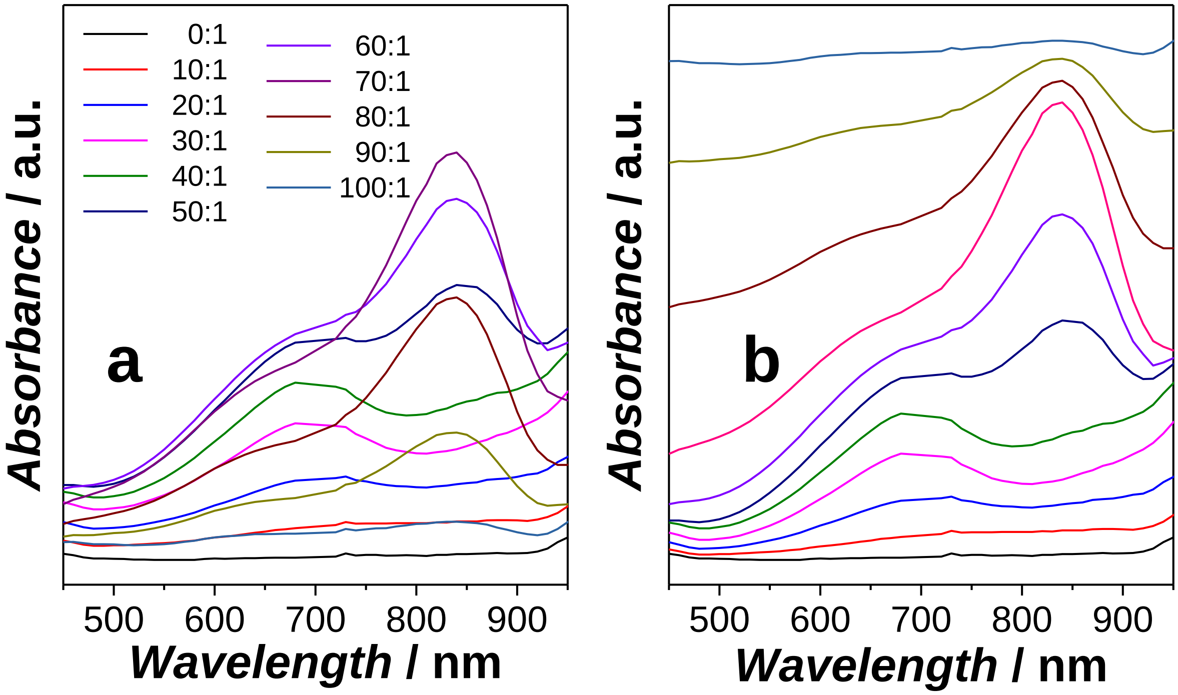

Supplementary Fig. 4 *Absorbance spectra of the PMA-LA mixture containing different LA:PMA Molar ratios (same PMA concentration with increasing LA) after UVA irradiation for 60 min showing (a) experimentally-recorded absorbance profile, and (b) absorbance profiles after vertical translation of data presented in (a) for clarity.*

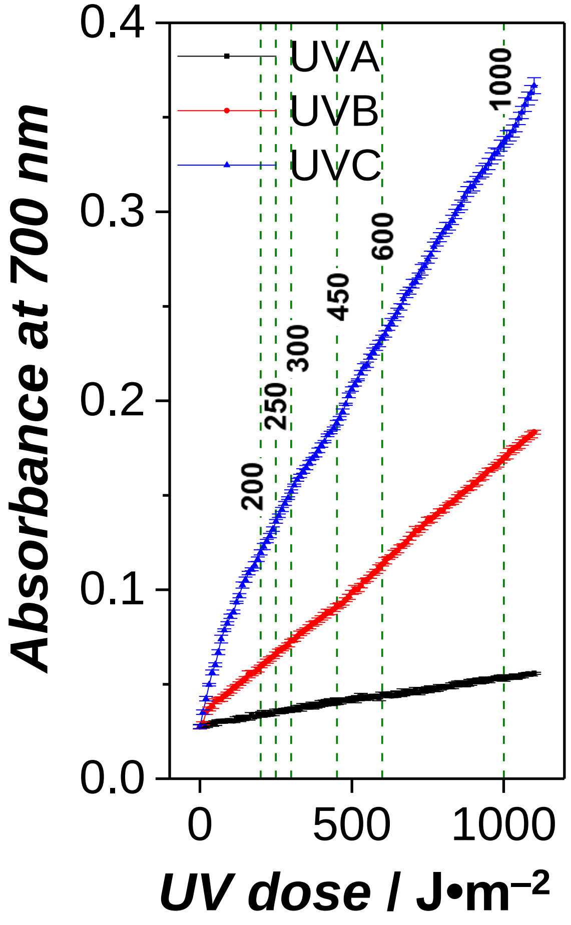

Supplementary Fig. 5 *The colorimetric response of PMA-LA sensor in the solution format, as discussed in the Fig.3a in the main manuscript, however with a linear X-axis in the current case. Each data point represents an average of the colorimetric response obtained from 12 independent sensors and the associated standard deviation.*

UV dose ($J \cdot m^{-2}$)	0	25	50	100	150	200	250	300	450	600	1000	1500	3000	4500	9000	18000
**Time (s)**	0	5	10	20	30	40	50	60	90	120	200	300	600	900	1800	3600

UVA

UVB

UVC

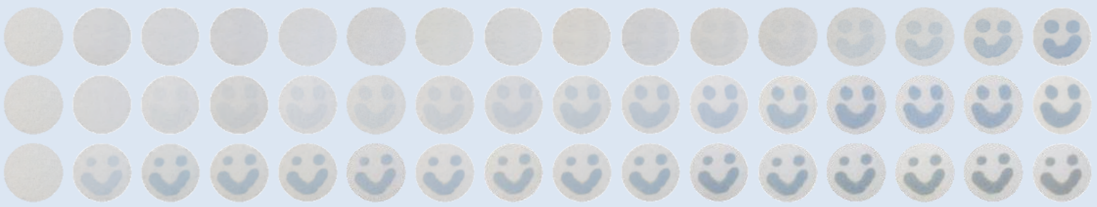

Supplementary Fig. 6 *Photographs of three paper-based UV sensors with increasing exposure time/ dose of UVA, B, and C. The photographs presented here are those as-captured using a mobile camera, whereas the edited version of these photographs is shown in Fig. 4a in the main manuscript.*

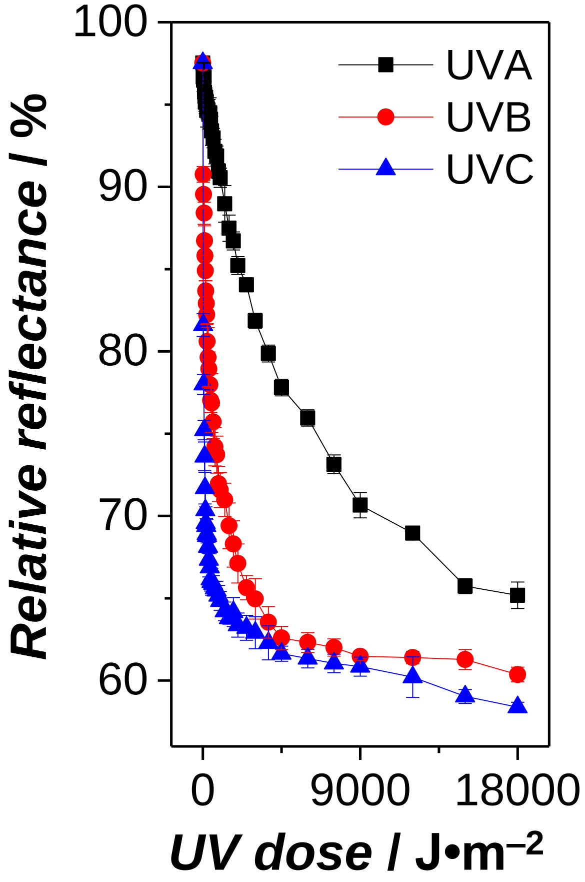

Supplementary Fig. 7 *The colorimetric response of PMA-LA sensor in the paper-based smiley format, as discussed in the Fig. 4e in the main manuscript, however with a linear X-axis in the current case. Each data point represents an average of the colorimetric response obtained from 4 independent sensors and associated standard deviation.*

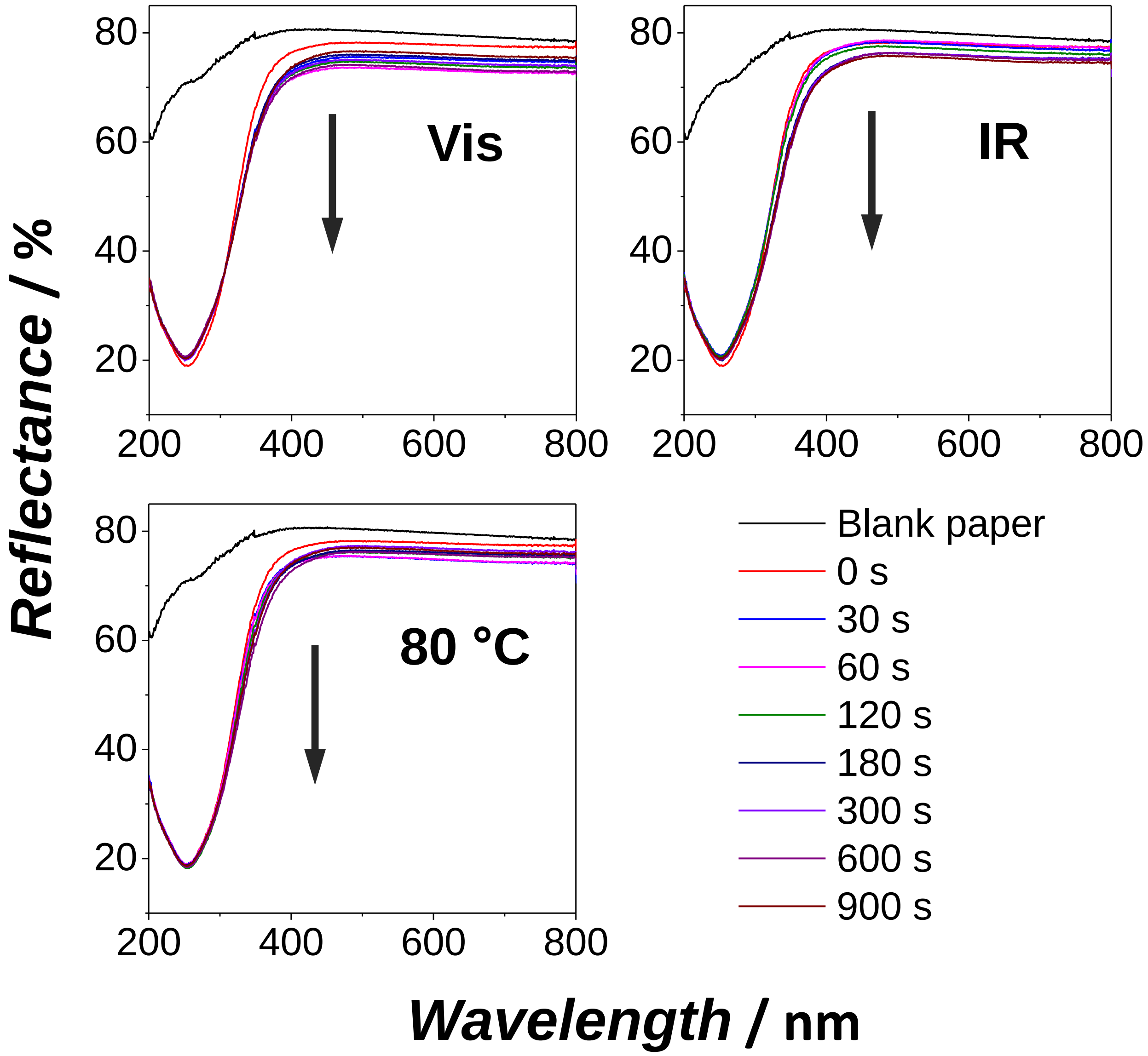

***Supplementary Fig. 8** Validation of the specificity of paper-based smiley sensors through studying their reflectance spectra under different exposure conditions, including high intensity visible light (130 W·m^{-2}), infrared light (700 W·m^{-2}) and 80 °C heat over a period of time.*

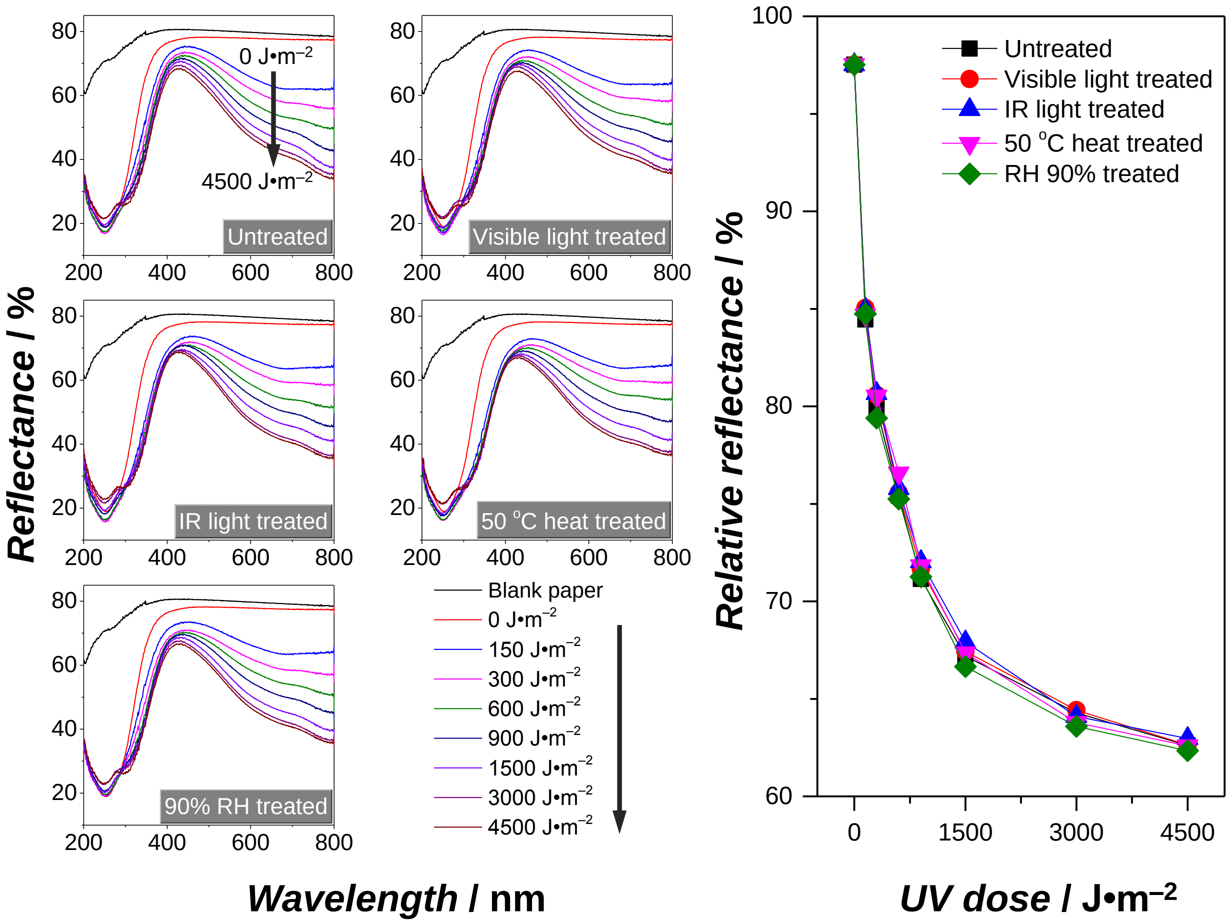

Supplementary Fig. 9 *Validation of the durability of paper-based smiley sensors through pre-exposing to different ambient-mimicking environmental conditions for one hour, followed by studying the changes in their reflectance spectra with increasing UVB dose, as indicated. The right panel compares the relative reflectance of different pre-treated smiley sensors at each of the tested UVB dose points.*

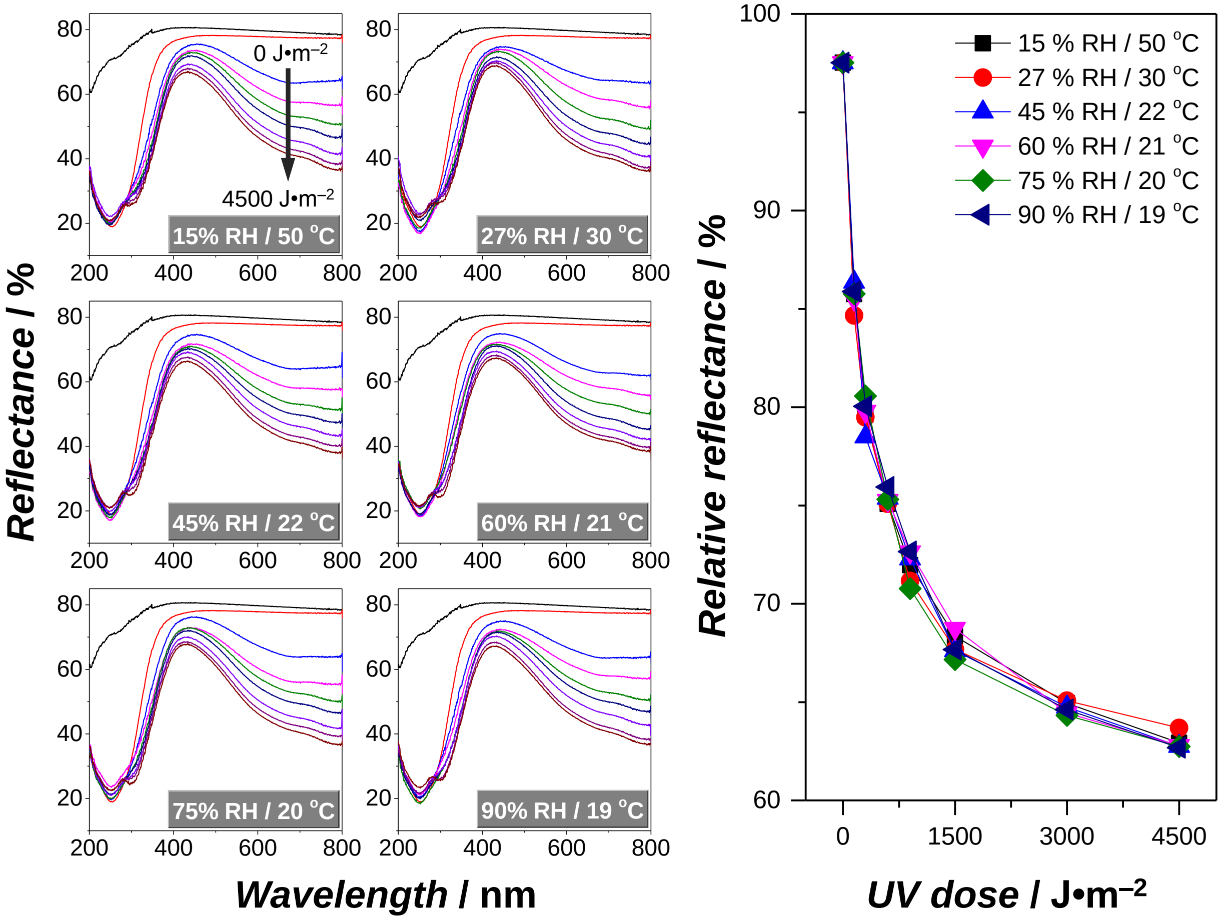

Supplementary Fig. 10 *Validation of the durability of paper-based smiley sensors through assessing their UVB sensing performance while simultaneously exposing them to a wide range of relative humidity and ambient temperature conditions. The right panel compares the relative reflectance of these smiley sensors at each of the tested UVB dose points.*

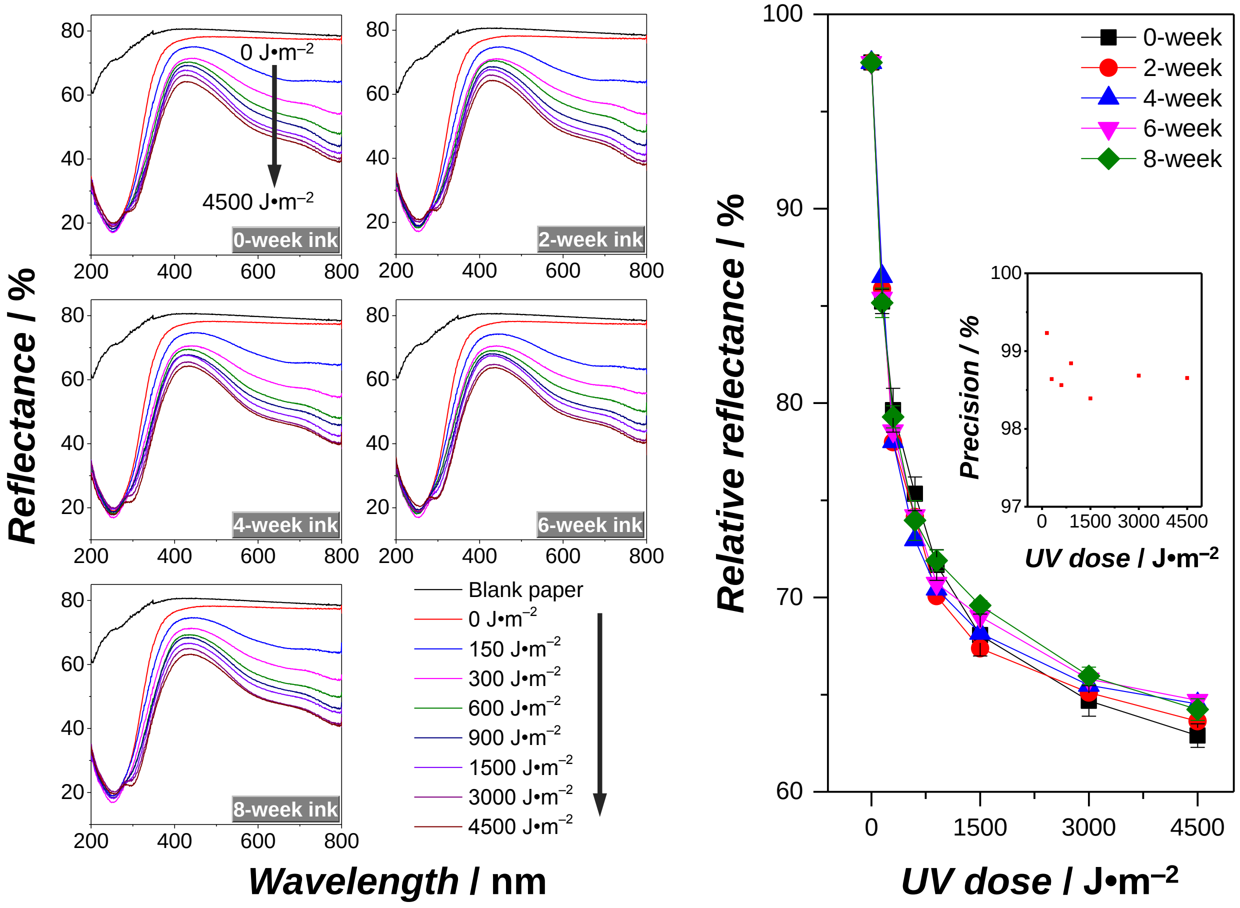

Supplementary Fig. 11 *Validation of the stability of PMA-LA ink through preparing the ink up to 8 weeks in advance, followed by fabricating the paper-based smiley sensors at 0th day, and assessing the dose-dependent UVB sensing performance of these paper-based sensors. The right panel compares the relative reflectance of these smiley sensors at each of the tested UVB dose points, and the inset shows the overall precision of these sensors. The data used for calculating precision includes responses from 25 independent sensors. These include 17 sensors prepared using fresh ink, 4 sensors prepared using 8-week old ink, and 1 sensor each prepared using 2-, 4- and 6-week old inks. The 17 sensors prepared using fresh ink had undergone different treatment conditions as outlined from data in Supplementary Fig. 9 (5 sensors), Supplementary Fig. 10 (6 sensors) and Fig. 4 in the main manuscript (4 sensors) along with 2 new sensors prepared during the stability study shown in Supplementary Fig. 11.*

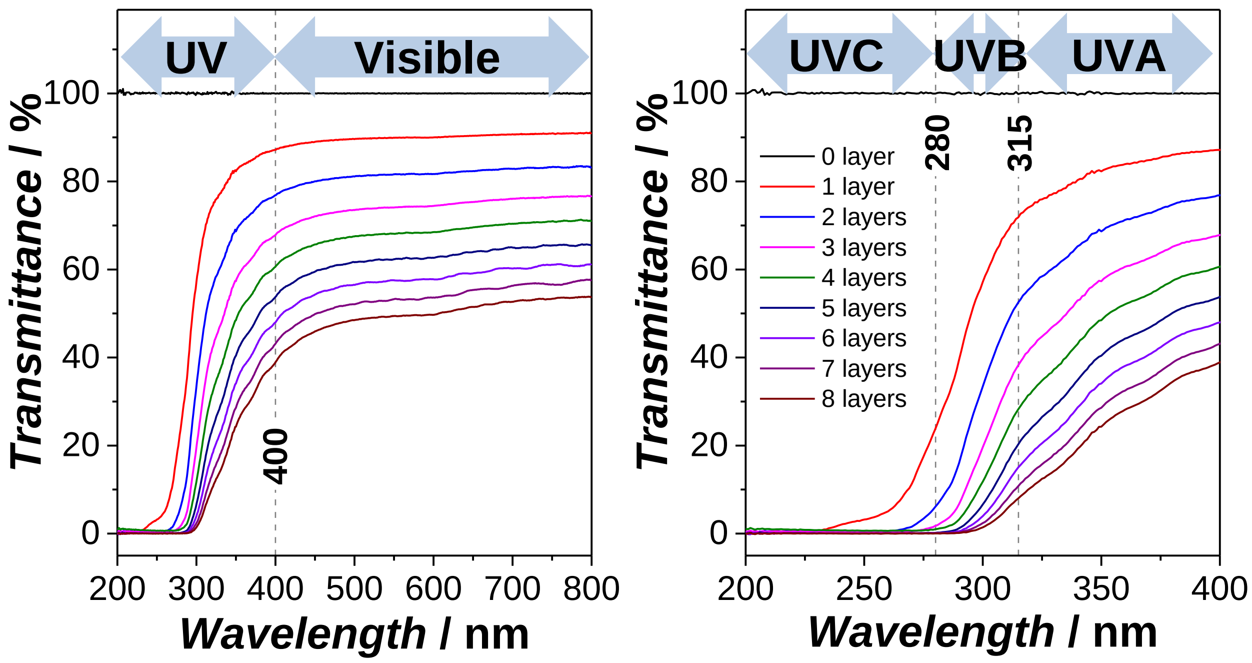

Supplementary Fig. 12 *Transmittance spectra of transparency film filters (TFF) containing different layers (0–8) of transparency sheets. The right panel shows the expanded UV region of spectra to clearly demonstrate the ability of TFF in blocking UV radiations.*

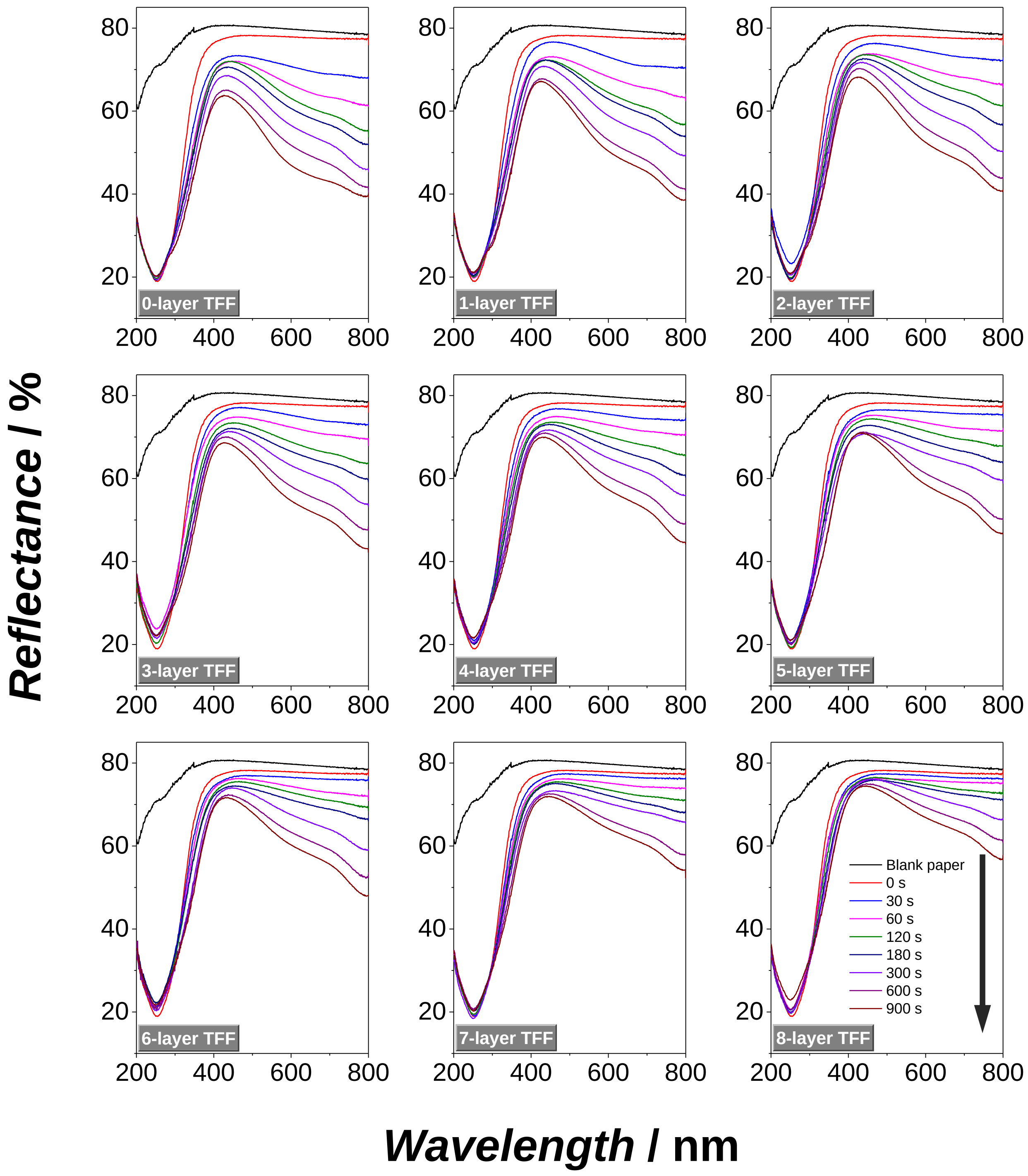

Supplementary Fig. 13 *Reflectance spectra of paper-based smiley sensors prepared using invisible PMA-LA ink after coating them with different layers of TFF, followed by exposure to simulated solar light for different time points. In these experiments, 1 s of solar irradiation corresponds to 1 J·m*$^{-2}$ *equivalent of UVB exposure to the sensors.*

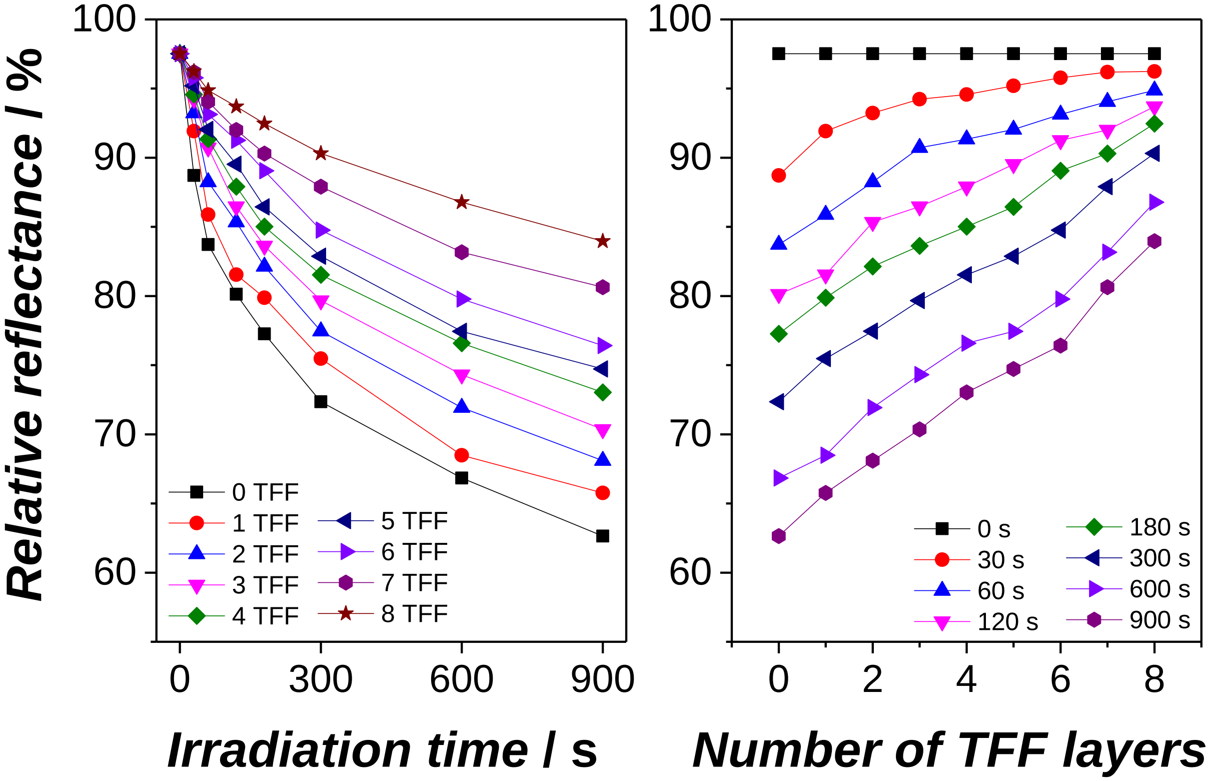

Supplementary Fig. 14 *Correlation between the numbers of TFF employed to prepare paper-based smiley sensors and the relative reflectance from blue smileys generated on exposure to the solar simulator. The left panel shows that irrespective of the number of TFF employed to prepare a particular sensor, the sensor continues to show time/ dose-dependent response. This is critical for this system to behave as a dosimeter. The right panel shows that when the sensor is exposed to a fixed amount of simulated solar light (each spectrum represents a fixed amount of solar UV dose), the sensor response can be delayed by increasing the number of TFF in the sensor. This is evident from the first order kinetic rate constants obtained from these data for different layers of TFF (0 layer: 0.0053 s^{-1}; 1 layer: 0.0048 s^{-1}; 2 layers: 0.0041 s^{-1}; 3 layers: 0.0039 s^{-1}; 4 layers: 0.0037 s^{-1}; 5 layers: 0.0033 s^{-1}; 6 layers: 0.0028 s^{-1}; 7 layers: 0.0025 s^{-1}; 8 layers: 0.0012 s^{-1}). This aspect is critical for modulating the sensor response under a particular set of ambient conditions by simply coating them with different layers of TFF. In these experiments, 1 s of solar irradiation corresponds to 1 $J{\cdot}m^{-2}$ equivalent of UVB exposure.*
